# Response to letter by Lo *et al.*: Investigating seasonal association between vitamin D concentration, muscle mass and strength in postmenopausal women: a critical analysis

**DOI:** 10.1017/jns.2023.34

**Published:** 2023-07-13

**Authors:** Anneka Welford, Andrea L. Darling, Sarah Allison, Susan A. Lanham-New, Carolyn A. Greig

**Affiliations:** 1 Leicester Diabetes Centre, University of Leicester, Leicester, UK; 2 Department of Nutritional Sciences, University of Surrey, Guildford, Surrey GU2 7XH, UK; 3 Biosciences & Medicine, University of Surrey Ringgold Standard Institution, Guildford, Surrey GU2 7XH, UK; 4 School of Sport, Exercise and Rehabilitation Sciences, MRC-Arthritis Research UK Centre for Musculoskeletal Ageing Research, University of Birmingham, Birmingham, UK

We would like to thank Lo *et al.* for their insightful commentary on our original article, ‘*Lack of significant seasonal association between serum 25(OH)D concentration, muscle mass and strength in postmenopausal women from the D-FINES longitudinal study*’.

The main focus of our analysis was to investigate the association between vitamin D status and muscle function in postmenopausal women, rather than factors affecting vitamin D status *per se*. That being said, we agree with the authors that individual variations in each of the factors they have listed, influence serum 25(OH)D concentration to a certain degree, and therefore could account for some of the variability in both the serum 25(OH)D and muscle function outcome data which we present.

Although we did not account for dietary or genetic factors within this analysis, some effort was made to describe UVB exposure, using proxy measures such as ethnicity and seasonal physical activity.

For example, we performed a basic description of the ethnicity of the participants and also completed a subgroup analysis of British white *v.* British Asian women. From this analysis, we found that postmenopausal British Asian women presented with significantly lower 25(OH)D concentrations compared with White British/Irish women and could be classified as vitamin D deficient during each season (summer: 28⋅41 nmol/l *v.* 61⋅64 nmol/l, autumn: 21⋅45 nmol/l *v.* 55⋅30 nmol/l, winter: 21⋅11 nmol/l *v.* 41⋅91 nmol/l, spring: 22⋅63 nmol/l *v.* 45⋅23 nmol/l, all *P* < 0⋅001). Our analysis also showed that British Asian women were weaker than White British/Irish women at all seasonal timepoints, statistically evident during the winter and spring.

This is consistent with the assumption that skin pigmentation influences serum vitamin D status, and a previous study has shown that British South Asian adults living within the UK may require more sun exposure to produce the same amount of vitamin D in the skin as their White British counterparts^(1)^. However, this effect is multifactorial and cannot be purely attributed to skin pigmentation alone; it is also further influenced by cultural factors, dietary and supplemental intake of vitamin D^(2)^.

Additionally, we assessed the physical activity of our participants as estimates of the number of hours per day spent in sleep, light, moderate and active work and leisure time activity in PALs. As with ethnicity, this could be considered a proxy measurement of UVB exposure.

The average self-reported daily walking time varied seasonally within the D-FINES cohort, with a trend towards more time spent walking in the summer. It is possible that the seasonal physical activity variation may have influenced cutaneous production of vitamin D, leading to higher serum concentrations evident within the summer months; this in turn may have driven the changes in muscle outcomes, although it was not possible to demonstrate this.

In conclusion, individual variations in the effects of diet, sunlight exposure/absorption and genotype, as well as exposure and engagement in physical activity could account for some of the variability in both the vitamin D and muscle function outcome data, and we agree with Lo *et al.*, in this regard.

We also accept that it would be important to include these in future analyses investigating seasonal changes in vitamin D status as this would give us more confidence in attributing changes in vitamin D concentration as ‘truly’ seasonal.
